# Iso-Oncotic Albumin Mitigates Brain and Kidney Injury in Experimental Focal Ischemic Stroke

**DOI:** 10.3389/fneur.2020.01001

**Published:** 2020-09-03

**Authors:** Renata de S. Mendes, Gloria Martins, Milena V. Oliveira, Nazareth N. Rocha, Fernanda F. Cruz, Mariana A. Antunes, Soraia C. Abreu, Adriana L. Silva, Christina Takiya, Pedro M. Pimentel-Coelho, Chiara Robba, Rosália Mendez-Otero, Paolo Pelosi, Patricia R. M. Rocco, Pedro L. Silva

**Affiliations:** ^1^Laboratory of Pulmonary Investigation, Institute of Biophysics Carlos Chagas Filho, Federal University of Rio de Janeiro, Rio de Janeiro, Brazil; ^2^Laboratory of Imunophysiology, Institute of Biophysics Carlos Chagas Filho, Federal University of Rio de Janeiro, Rio de Janeiro, Brazil; ^3^Laboratory of Cellular and Molecular Neurobiology, Institute of Biophysics Carlos Chagas Filho, Federal University of Rio de Janeiro, Rio de Janeiro, Brazil; ^4^San Martino Policlinico Hospital, IRCCS for Oncology and Neurosciences, University of Genoa, Genoa, Italy; ^5^Department of Surgical Sciences and Integrated Diagnostics (DISC), University of Genoa, Genoa, Italy; ^6^Rio de Janeiro Network on Neuroinflammation, Carlos Chagas Filho Foundation for Supporting Research in the State of Rio de Janeiro (FAPERJ), Rio de Janeiro, Brazil

**Keywords:** stroke, hemodynamic, albumin, inflammation, kidney damage

## Abstract

**Background:** There is widespread debate regarding the use of albumin in ischemic stroke. We tested the hypothesis that an iso-oncotic solution of albumin (5%), administered earlier after acute ischemic stroke (3 h), could provide neuroprotection without causing kidney damage, compared to a hyper-oncotic albumin (20%) and saline.

**Objective:** To compare the effects of saline, iso-oncotic albumin, and hyper-oncotic albumin, all titrated to similar hemodynamic targets, on the brain and kidney.

**Methods:** Ischemic stroke was induced in anesthetized male Wistar rats (*n* = 30; weight 437 ± 68 g) by thermocoagulation of pial blood vessels of the primary somatosensory, motor, and sensorimotor cortices. After 3 h, animals were anesthetized and randomly assigned (*n* = 8) to receive 0.9% NaCl (Saline), iso-oncotic albumin (5% ALB), and hyper-oncotic albumin (20% ALB), aiming to maintain hemodynamic stability (defined as distensibility index of inferior vena cava <25%, mean arterial pressure >80 mmHg). Rats were then ventilated using protective strategies for 2 h. Of these 30 animals, 6 were used as controls (focal ischemic stroke/no fluid).

**Results:** The total fluid volume infused was higher in the Saline group than in the 5% ALB and 20% ALB groups (mean ± SD, 4.3 ± 1.6 *vs*. 2.7 ± 0.6 and 2.6 ± 0.5 mL, *p* = 0.03 and *p* = 0.02, respectively). The total albumin volume infused (g/kg) was higher in the 20% ALB group than in the 5% ALB group (1.4 ± 0.6 *vs*. 0.4 ± 0.2, *p* < 0.001). Saline increased neurodegeneration (Fluoro-Jade C staining), brain inflammation in the penumbra (higher tumor necrosis factor-alpha expression), and blood-brain barrier damage (lower gene expressions of claudin-1 and zona occludens-1) compared to both iso-oncotic and hyper-oncotic albumins, whereas it reduced the expression of brain-derived neurotrophic factor (a marker of neuroregeneration) compared only to iso-oncotic albumin. In the kidney, hyper-oncotic albumin led to greater damage as well as higher gene expressions of kidney injury molecule-1 and interleukin-6 than 5% ALB (*p* < 0.001).

**Conclusions:** In this model of focal ischemic stroke, only iso-oncotic albumin had a protective effect against brain and kidney damage. Fluid therapy thus requires careful analysis of impact not only on the brain but also on the kidney.

## Introduction

In ischemic stroke, maintenance of appropriate systemic perfusion is an important therapeutic goal (1). Fluids are often administered to achieve hemodynamic targets; however, there are controversies regarding the effects of different types of fluids on brain as well as kidney function. Even though normal saline (NaCl 0.9%) has been recommended in ischemic stroke (2, 3), it may lead to distal organ damage in critically ill patients (4–6).

Experimental studies have shown that the administration of hyper-oncotic (25%) albumin may have neuroprotective effects on brain ischemia by reducing infarct volume and cerebral edema (7, 8). Nevertheless, the potential benefits of hyper-oncotic albumin have not been confirmed in clinical trials (9, 10). The ALIAS II trial, which compared hyper-oncotic albumin (25%) with an equivalent volume of isotonic saline infusion within 5 h after onset of clinical signs of acute ischemic stroke, was stopped prematurely for futility (9). Since then, hyper-oncotic albumin has not been recommended for the treatment of patients with ischemic stroke (2, 3). Nevertheless, certain aspects that require special attention, such as: (1) the timing of albumin administration, which may be an important variable for which to control (11)—the earlier the intervention, the better the outcome might be; and (2) hyper-oncotic albumin may be associated with kidney damage proportional to its concentration, thus worsening prognosis (12, 13). Based on the foregoing, we tested the hypothesis that an iso-oncotic solution of albumin (5%), administered earlier after acute ischemic stroke (3 h), could provide neuroprotection without causing kidney damage, compared to a hyper-oncotic albumin (20%) and saline. Therefore, the effects of saline, 5% albumin, and 20% albumin were evaluated on neurodegeneration, blood-brain barrier permeability, as well as brain and kidney damage in experimental focal ischemic stroke.

## Methods

### Study Approval

This study was approved by the Ethics Committee of the Carlos Chagas Filho Institute of Biophysics (CEUA 100/18), Federal University of Rio de Janeiro, Rio de Janeiro, Brazil. All animals received humane care in compliance with the “Principles of Laboratory Animal Care” formulated by the National Society for Medical Research and the *Guide for the Care and Use of Laboratory Animals* prepared by the National Academy of Sciences, USA. The present study followed the ARRIVE guidelines for reporting of animal research (14). Animals were housed at a controlled temperature (23°C) and controlled light–dark cycle (12–12 h), with free access to water and food, while no acclimation was done. Animals were not specific-pathogen-free (SPF).

### Animal Preparation and Experimental Protocol

Thirty male Wistar rats (boy weight 437 ± 68 g) were anesthetized with xylazine 2.5 mg/kg intraperitoneally (i.p.) and ketamine 75 mg/kg i.p. and placed in a stereotactic frame with their heads immobilized. Animals were subjected to ischemic stroke induction by thermocoagulation of the pial blood vessels overlying the primary somatosensory, motor, and sensorimotor cortices (15–17). Briefly, the skull was surgically exposed, and a craniotomy performed, exposing the left frontoparietal cortex (+2 to−6 mm A.P. from bregma). Blood in pial vessels was thermocoagulated transdurally by approximation of a hot probe to the dura mater with temperature set to 300°C (18). The color of the pial vessels is normally light red; blood was considered thermocoagulated once it had turned dark red within 5 min. After the overall procedure, which lasted 40 min, the operative wound was closed, animals were kept warm under a heating lamp (rectal temperature 37.5 ± 1°C), and returned to their cages after recovery from anesthesia. The stroke procedure was always performed by the same experienced investigator (R.S.M.) in order to minimize differences in stroke severity.

Of these 30 animals, 6 were used as controls (focal ischemic stroke/no fluid) for molecular biology analysis. The focal ischemic stroke model avoids damage to hypothalamus, hippocampus and midbrain, and offers the possibility to promote reperfusion of the ischemic brain tissue as compared to intraluminal suture middle cerebral artery occlusion models (16). Moreover, this model of ischemic stroke induces forelimb motor asymmetry, evidence of cerebral infarction on magnetic resonance imaging, and changes in systolic peak velocity by carotid Doppler ipsilateral to the injury (19).

**Figure 1:**
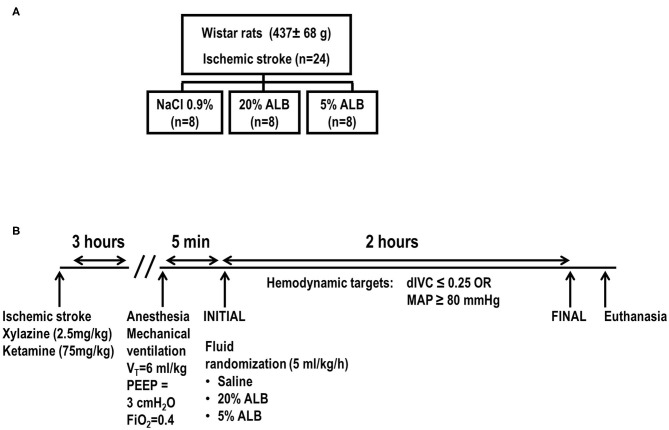
Study randomization **(A)** and experimental design **(B)**. Schematic illustration of the model of focal ischemic stroke by craniectomy and thermocoagulation of pial vessels over the right primary sensorimotor cortex is shown in the bottom left. dIVC, distensibility index of inferior vena cava; MAP, mean arterial pressure; V_T_, tidal volume; PEEP, positive end-expiratory pressure; FiO_2_, inspired fraction of oxygen; 5% ALB, 5% albumin; 20% ALB, 20% albumin.

After 3 h, following local subcutaneous anesthesia with 2% lidocaine (0.4 mL), a midline cervical incision and tracheostomy were performed. An intravenous (i.v.) catheter (Jelco® 24G, Becton, Dickinson and Company, USA) was inserted into the tail vein, and anesthesia was induced and maintained with midazolam (2 mg/kg/h) and ketamine (50 mg/kg/h). The adequacy of anesthesia was assessed by response to a nociceptive stimulus before surgery. A second catheter (18G; Arrow International, USA) was then placed in the right internal carotid artery for blood sampling and arterial blood gas analysis (ABL80 FLEX; Radiometer Medical, Denmark), as well as for monitoring of MAP and rectal temperature continuously (Networked Multiparameter Veterinary Monitor LifeWindow 6000V; Digicare Animal Health, Florida, USA). Rectal temperature was kept constant at 37.5 ± 1°C using a heating pad during stroke induction and mechanical ventilation. Animals were paralyzed with 2 mg/kg pancuronium bromide (Pancuron®, Cristália, Itapira, SP, Brazil) and mechanically ventilated (Servo-i, MAQUET, Switzerland) in volume-controlled mode with tidal volume (*V*_T_) = 6 mL/kg, respiratory rate (RR) to maintain normocapnia (PaCO_2_ = 35–45 mmHg), fraction of inspired oxygen (FiO_2_) = 0.4, and positive end-expiratory pressure (PEEP) = 3 cm H_2_O. Rats were then randomly assigned by computer sequence to one of three groups (*n* = 8/group) (Figure 1A): (1) normal saline (0.9% NaCl); (2) 5% ALB; called iso-oncotic fluid; or (3) 20% ALB; called hyper-oncotic fluid (ALBUREX®, CSL Behring AG, Bern, Switzerland). Fluids were infused through the tail vein using a different three-way as that used for anesthesia and adapted through the intravenous catheter (Jelco® 24G, Becton, Dickinson and Company, USA). Detailed descriptions of the composition of each fluid are provided in Table S1. Functional data were obtained immediately after randomization (INITIAL) and at the end of the experiment, i.e., after 2 h of mechanical ventilation (FINAL). At FINAL, heparin (1000 IU) was injected into the tail vein and animals were euthanized by overdose of sodium thiopental (150 mg/kg). The brain and kidneys were carefully removed for histology and molecular biology analysis (Figure 1B).

### Maintenance of Hemodynamic Stability

During mechanical ventilation, hemodynamic stability was maintained by evaluating the distensibility index of the inferior vena cava (dIVC) every 30 min and MAP continuously. Our group has previously validated the dIVC for small animals (20); according to our protocol, fluid infusion (5 mL/kg/h) was administered when dIVC values were > 0.25 and stopped when lower values were achieved. However, if MAP remained <80 mmHg even after dIVC had fallen below 0.25, a fluid bolus (2 mL/kg) was infused. In short, the hemodynamic targets were dIVC <0.25 and MAP >80 mmHg.

### Transthoracic Echocardiography

Transthoracic echocardiography was performed by a single, experienced examiner (N.N.R.), blinded to group allocation, using an UGEO HM70A system (Samsung, São Paulo, Brazil) equipped with a linear phased-array probe (8–13 MHz). The dIVC and stroke volume (SV) were evaluated. dIVC was calculated as (maximum diameter on inspiration - minimum diameter on expiration)/minimum diameter on expiration. Immediately after each evaluation of the IVC, the same transducer was moved to the left anterior chest wall for quantification of left ventricular SV. To reduce any error in the measurement of SV and dIVC, this procedure lasted <1 min. SV was obtained by multiplying the cross-sectional area of the aorta (measured in a B-mode parasternal long-axis view of the heart) by its velocity time integral (VTI). VTI was calculated by pulsed-wave Doppler analysis of the left ventricle outflow tract (21, 22). Heart rate (HR) was assessed from the subcostal view.

### Histology

#### Neurodegeneration by Immunofluorescence

Fluoro-Jade C was used to evaluate the degree of neurodegeneration in perilesional areas of the brain. This technique stains all degenerating neurons, regardless of the specific insult or mechanism of cell death. Brains were removed and fixed in 10% formalin for 18 h at 4°C. They were then cut coronally into 5-mm sections, processed in paraffin, cut serially at 6 μm thickness on a rotary microtome, and mounted on positively charged slides. After drying at room temperature for 24 h, sections were dewaxed in xylene, hydrated, kept in an overnight protein bath (5% bovine albumin in phosphate-buffered saline) to minimize autofluorescence, and permeabilized with 0.5% Triton-X100 in phosphate-buffered saline for 15 min. For Fluoro-Jade C staining, a slightly modified version of the protocol established by Schmued et al. (23) was used. Sections were then immersed in 70% ethanol for 2 min, followed by a bath in 1% NaOH in 80% ethanol for 5 min, and rinsed consecutively in 70% ethanol and distilled water (2 min each). Then, sections were incubated in 0.06% potassium permanganate for 15 min, rinsed again in distilled water (1 min), and transferred to a 0.0001% solution of Fluoro-Jade C in 0.1% acetic acid, freshly prepared from aqueous stock solution (0.01%), for 15 min. Finally, sections were rinsed in distilled water, mounted with acid glycerin mounting medium, and observed in an epifluorescence microscope (Eclipse E800, Nikon, Japan).

Semiquantitative data were derived from high-resolution photomicrographs (2,048 × 1,536 pixel buffer) acquired using a digital camera (Evolution VF, Media Cybernetics Inc., Bethesda, MD, USA) connected to a Nikon Eclipse E-800 light microscope and Q-capture 2.95.0 software, version 2.0.5 (Silicon Graphic Inc., Milpitas, CA, USA). Ten non-overlapping images of perilesional brain areas were taken. Quantification of Fluoro-Jade C positive cells was performed by one investigator (C.T.) and monitored by a second investigator (M.V.O.) blinded to group assignment. Data are shown as degenerated cells per perilesional brain area (cells/mm^2^).

#### Kidney Damage

Right kidney sections were examined by a pathologist blinded to group allocation (C.T.). Paraffin sections (6 μm) were stained with hematoxylin–eosin (H&E) and periodic acid–Schiff reactive (PAS) and observed under light microscopy for qualitative and quantitative analysis. “Tubular injury” was defined as tubular cell injury characterized by absence/derangement of the brush border in proximal tubular epithelia, tubular cell vacuolization, tubular cell death/desquamation, tubular obstruction due to the presence of cellular debris or casts leading to tubular dilation, and (later in the course of injury) tubular atrophy due to loss of viable tubular cells. Semiquantitative data were obtained from high-resolution photomicrographs, as described above for brain tissue imaging. Fifteen non-overlapping images of tubular tissue (cortex and outer medulla) were randomly obtained with a × 40 objective lens, from each kidney section (*n* = 8/group) stained with PAS (tubular profiles). Histological findings were graded from 0 to 4 (0, no change; 1, changes affecting 25% of the field of view; 2, changes affecting 25–50%; 3, changes affecting 51–75%, and 4, changes affecting >75% of the field), according to the area affected by the features of interest (absent/deranged brush border in proximal tubular epithelia, tubular cell vacuolization, tubular cell death/desquamation, tubular obstruction due to presence of cellular debris or casts). The final score in each rat was expressed as the sum of all values of all features obtained, and ranged from 0 to 20. Blinded analyses were carried out by two investigators (M.V.S.F. and A.L.S.).

### Biological Markers in Brain and Kidney Tissue

Perilesional brain area RNA was isolated from the same formaldehyde-fixed, paraffin-embedded tissue used for H&E. The MagMAX™ FFPE DNA/RNA Ultra Kit (ThermoFisher Scientific) was used for the brain, while the RNeasy Plus Mini Kit (Qiagen, Germany) was used for frozen left kidney tissue. Following the manufacturer's instructions, downstream amplification assays targeting short PCR amplicons (200 nucleotides or less) were designed. Additionally, to avoid contaminating RNases, sterile gloves, disposable tubes, and aerosol-resistant pipette tips were used at all times. RNA was recovered and quantitative real-time reverse transcription polymerase chain reaction (RT-PCR) was performed to measure biomarkers in perilesional areas of brain tissue. Gene expressions of biomarkers associated with inflammation (tumor necrosis factor alpha [TNF-α]), neuroregeneration (brain-derived neurotrophic factor [BDNF]), and blood-brain barrier integrity (claudin-1 and zonula occludens-1 [ZO-1]) were evaluated. In left kidney tissue, gene expressions of biomarkers associated with renal injury (kidney injury molecule [KIM]-1) and inflammation (interleukin [IL]-6) were evaluated. The primers are shown in Table S2. For each sample, the expression of each gene was measured in triplicate and normalized to the acidic ribosomal phosphoprotein P0 (*36B4*) housekeeping gene (24) and expressed as fold change relative to Saline group, using the 2^−ΔΔ*Ct*^ method, where ΔCt = Ct_target gene_ – Ct_reference gene_ (25). Blinded analyses were carried out by one investigator (M.A.A.).

### Statistical Analysis

All efforts were made to minimize animal suffering and the number of animals used in this study. Six animals per group would provide the appropriate power (1–β = 0.8) to identify significant (*p* < 0.05) differences in brain damage (the primary endpoint) between saline and albumin during early therapy after ischemia, according to previous studies (26) and pilot studies using 20% ALB, taking into account an effect size *d* = 2.0, a two-sided test, and a sample size ratio = 1 (G^*^Power 3.1.9.2; University of Düsseldorf, Düsseldorf, Germany).

The Kolmogorov–Smirnov test with Lilliefors' correction was used to assess normality of data, while the Levene median test was used to evaluate the homogeneity of variances. Functional data obtained at INITIAL and FINAL were assessed by two-way ANOVA followed by Holm–Šídák's multiple comparisons to compare parameters among groups and between time points (INITIAL and FINAL). Molecular biology variables and histological score data were compared using the Kruskal–Wallis test followed by Dunn's multiple comparisons. Parametric data were expressed as mean ± SD, and non-parametric data, as median (interquartile range). All tests were performed in GraphPad Prism version 6.00 (GraphPad Software, La Jolla, CA, USA). Significance was established at *p* < 0.05.

## Results

The survival rate was 100% in all groups, and there were no missing data. At FINAL, both hemodynamic targets (dIVC and MAP) were reached after fluid administration in all groups (Table 1). For achievement of the same hemodynamic targets, the total volume of fluid infused was higher in the Saline group than in 5% ALB and 20% ALB (*p* = 0.03 and *p* = 0.02, respectively). The total amount of albumin infused normalized by body weight (g/kg) in the 5% ALB group was lower than in the 20% ALB (0.42 ± 0.22 *vs*. 1.42 ± 0.56, *p* < 0.001). PaCO_2_, pHa, bicarbonate, chloride, and anion gap were similar among groups (Table S3). Although overall time effect was observed in PaCO_2_, no differences were detected within each group. No major differences were observed in respiratory system mechanics (Table S4).

**Table 1:** Total volume infused and cardiovascular parameters.

**Parameter**	**Group**	**Initial**	**Final**	**Time**	**Group**	**Time *vs*. group**
Total volume infused	Saline	—	4.3 ± 1.6	–	–	–
(mL)	5% ALB		2.7 ± 0.6*			
	20% ALB	—	2.6 ± 0.5*			
Albumin infused	Saline	—	–			
(g/kg)	5% ALB	—	0.42 ± 0.22			
	20% ALB	—	1.42 ± 0.56^†^	–	–	–
Hematocrit	Saline	0.38 ± 0.06.	0.38 ± 0.03			
(SI)	5% ALB	0.38 ± 0.05	0.34 ± 0.07	*p* = 0.10	*p* = 0.64	*p* = 0.18
	20% ALB	0.39 ± 0.03	0.38 ± 0.06			
MAP	Saline	72 ± 21	104 ± 17^‡^			
(mmHg)	5% ALB	78 ± 35	100 ± 20^‡^	*p* < 0.01	*p* = 0.78	*p* = 0.82
	20% ALB	77 ± 25	112 ± 36^‡^			
HR	Saline	318 ± 66	325 ± 66			
(bpm)	5% ALB	337 ± 91	351 ± 59	*p* = 0.70	*p* = 0.32	*p* = 0.46
	20% ALB	324 ± 71	280 ± 82			
dIVC	Saline	0.56 [0.42–0.83]	0.22 [0.14–0.29]^‡^			
(SI)	5% ALB	0.39 [0.33–0.84]	0.16 [0.09–0.25]^‡^	*p* < 0.001	*p* = 0.71	*p* = 0.99
	20% ALB	0.42 [0.34–0.90]	0.20 [0.05–0.25]^‡^			
SV	Saline	0.26 ± 0.12	0.33 ± 0.12^‡^			
(mL)	5% ALB	0.36 ± 0.14	0.42 ± 0.08^‡^	*p* = 0.01	*p* = 0.20	*p* = 0.19
	20% ALB	0.27 ± 0.05	0.54 ± 0.26^‡^			
CO	Saline	87 ± 53	97 ± 77			
(mL/min)	5% ALB	124 ± 40	132 ± 53	*p* = 0.20	p = 0.35	*p* = 0.59
	20% ALB	93 ± 26	140 ± 104^‡^			

*Data are shown as mean ± SD; 20% ALB, 20% albumin; 5% ALB, 5% albumin. Comparisons among groups at each time point (INITIAL and FINAL) were done by two-way ANOVA followed by Holm–Šídák's multiple comparisons. For comparison of total volume infused, the Kruskal–Wallis test with Dunn's multiple comparisons was used. For comparison of albumin volume infused, the Mann–Whitney U-test was used. MAP, mean arterial pressure; HR, heart rate; dIVC, distensibility index of the inferior vena cava; SV, stroke volume; CO, cardiac output; SI, International System of Units*.

* *vs Saline (p < 0.05)*;

† *vs 5% ALB (p < 0.01)*;

‡ *vs Initial (p < 0.05)*.

### Brain Histology and Biological Markers

**Figure 2:**
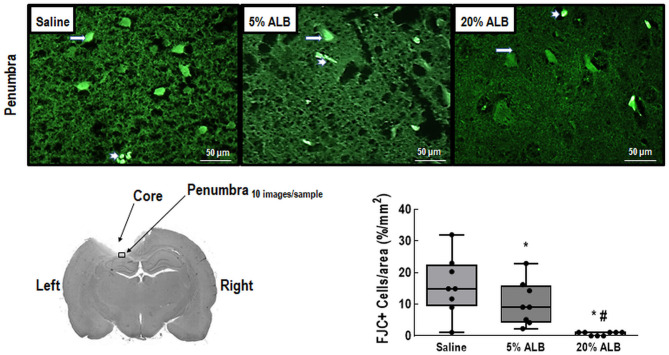
Comparison of Fluoro-Jade C (FJC) staining between Saline (0.9% NaCl), 5% ALB (albumin), and 20% ALB groups. Long arrows point to FJC-positive cells, and short arrows, to vessels. Note representative diagram of a brain slice after ischemic brain injury, indicating the core and penumbra regions. Within the penumbra, a sample area from which FJC images were taken is highlighted. Comparisons among groups were performed by the Kruskal–Wallis test followed by Dunn's test. * *vs*. Saline; # *vs*. 5% ALB. Significance accepted at *p* < 0.05.

**Figure 3:**
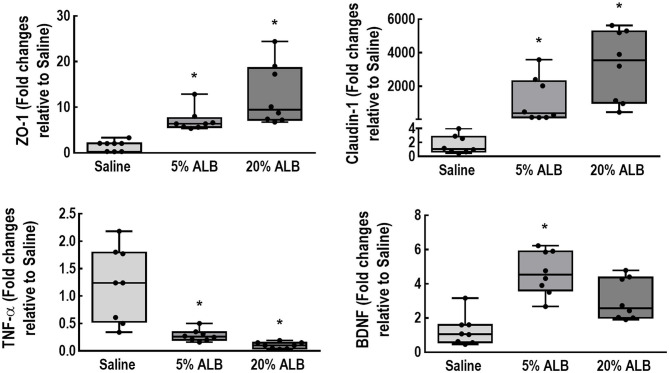
Expression of biologic markers associated with brain damage. Real-time polymerase chain reaction analysis of biologic markers associated with blood-brain barrier integrity (zonula occludens [ZO]-1 and claudin-1), brain inflammation (tumor necrosis factor [TNF]-α), and neurotrophic activity (brain-derived neurotrophic factor [BDNF]). Boxes show the interquartile range (25–75th percentile), while whiskers encompass the range (minimum-maximum) and horizontal lines represent the median in 8 animals/group. Scatter plots are also presented. Relative gene expression was calculated as a ratio of average expression of each gene to the reference gene (*36B4*) and expressed as fold change relative to Saline group. Comparisons among all groups were done using the Kruskal–Wallis test followed by Dunn's *post hoc* test. * *vs*. Saline. For all comparisons, significance accepted at *p* < 0.05.

Neurodegeneration, expressed by the number of Fluoro-Jade C-positive cells per field, was higher in Saline compared to the 5% ALB and 20% ALB groups (13.8 ± 6.0 *vs*. 9.0 ± 5.9 and 0.6 ± 0.5, respectively; *p* = 0.02 and < 0.001) (Figure 2). In brain tissue, mRNA expression of TNF-α was higher in the Saline than in the 5% ALB and 20% ALB groups (median [interquartile range], 1.24 [0.47–1.97] *vs*. 0.26 [0.19–0.35], and 0.10 [0.03–0.18], *p* = 0.04 and *p* = 0.002, respectively). BDNF was lower in Saline than in 5% ALB (1.03 [0.54–2.11] *vs*. 4.53 [3.08–5.85], *p* = 0.04). Claudin-1 mRNA expression was lower in Saline compared to 5% ALB and 20% ALB (0.9 [0.6–2.6] *vs*. 291 [132–2,019], and 3,550 [1,135–5,198], respectively, *p* = 0.04 and *p* = 0.002). A similar pattern was observed for ZO-1 mRNA expression (2.1 [0.3–2.7] *vs*. 6.4 [5.5–9.7], and 8.8 [6.9–20.8], respectively, *p* = 0.04 and *p* = 0.005), which suggests a greater impairment of blood-brain barrier integrity (Figure 3).

### Kidney Histology and Biological Markers

**Figure 4:**
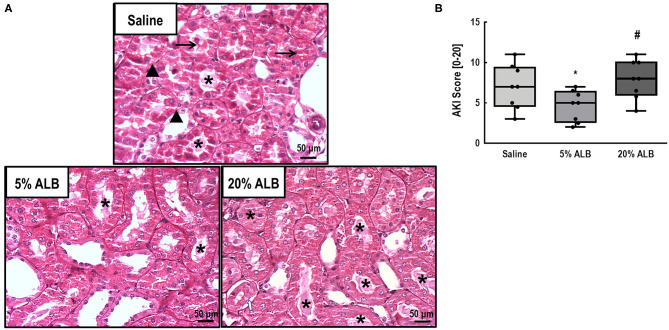
Acute kidney injury (AKI) score (absence of brush border, presence of vacuolization, desquamation, cell debris, and hyaline casts). **(A)**: Representative photomicrographs (light microscopy) of kidney tubules stained with periodic acid–Schiff reagent (PAS). Absence/derangement of the brush border in proximal tubular epithelia (▴), tubular cell vacuolization (→), and tubular obstruction due to the presence of cellular debris or casts (*) in kidney histological sections of 6–8 animals (original magnification, × 200). **(B)**: AKI score. Data are shown as median [interquartile range]. Comparisons among all groups were done using the Kruskal–Wallis test followed by Dunn's *post hoc* test. Five percentage ALB: 5% albumin; 20% ALB: 20% albumin. * *vs*. Saline; # *vs*. 5% ALB. For all comparisons, significance accepted at *p* < 0.05.

Kidney damage score was higher in 20% ALB compared to 5% ALB (*p* < 0.001) and Saline (*p* < 0.003), mainly due to absence of the brush border of tubular cells an d presence of vacuolization, desquamation, cell debris, and hyaline casts (Figure 4).

**Figure 5:**
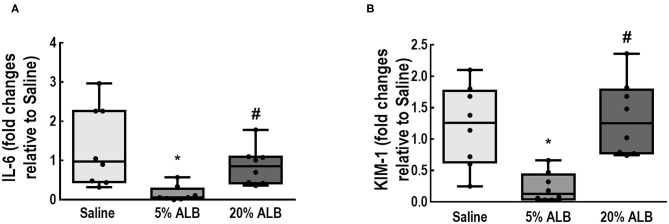
Expression of biologic markers associated with kidney damage. **(A)** Interleukin (IL)-6 mRNA levels; **(B)** Kidney injury molecule-1 (KIM-1) mRNA levels. Comparisons among all groups were done using the Kruskal–Wallis test, with significance accepted at *p* < 0.05. Boxes show the interquartile range (25–75th percentile), while whiskers encompass the range (minimum-maximum) and horizontal lines represent the median in 8 animals/group. Scatter plots are also presented. Relative gene expression was calculated as a ratio of average expression of each gene to the reference gene (*36B4*) and expressed as fold change relative to Saline group. * *vs*. Saline 0.9%; # *vs*. 5% ALB.

In kidney tissue, mRNA expression of KIM-1, an early marker of renal proximal tubular injury (27), was lower in 5% ALB than in Saline and 20% ALB (*p* = 0.019 and *p* = 0.005, respectively). Additionally, IL-6 mRNA expression, which denotes inflammation, was lower in 5% ALB than in Saline and 20% ALB (*p* = 0.010 and *p* = 0.047, respectively) (Figure 5).

## Discussion

Three hours after the experimental induction of focal ischemic stroke, we found that: (1) albumin solutions require a lower volume compared to Saline to achieve the same hemodynamic target; (2) 5% ALB and 20% ALB, compared to Saline, reduced the expression of genes associated with neurodegeneration, brain inflammation, and damage to the blood-brain barrier; and (3) 20% ALB resulted in greater kidney damage compared to 5% ALB and Saline. Thus, our data suggest that iso-oncotic albumin seems to have beneficial effects on brain injury 3 h after an ischemic insult, without being associated with kidney damage.

Focal ischemic stroke was induced by vessel thermocoagulation in the primary motor cortex. After 24 h, this model has been shown to lead to sensorimotor dysfunction (17), local inflammation, and blood-brain barrier damage (19, 28). In a middle cerebral artery occlusion model, reperfusion occurs in both the ischemic core and penumbra, whereas focal ischemic stroke by vessel thermocoagulation permits reperfusion of the penumbra alone. This period of analysis (3 h after ischemia) was chosen since it is regarded as the therapeutic window used in clinical practice for interventions, such as thrombolysis. During this period, therapeutic strategies may have substantial impact on outcome (29). In our study, two different hemodynamic targets were used: dIVC <0.25 (20) and MAP > 80 mmHg, the latter recommended by recent studies (1, 3). A greater volume of saline solution than of albumin (~1.6 times as much) was required to achieve the same hemodynamic targets, regardless of albumin concentration. This finding is in agreement with previously published data (30), and confirms the role of albumin as a volume expander with a greater T½ than the crystalloids (31). There was no difference in the total volume infused between iso- and hyperoncotic albumin; this can be attributed to the clearance of albumin in rodents, which can reach up to 50% in 6 h (32).

In this model of focal stroke, we found that, compared to Saline, both albumin concentrations reduced neurodegeneration (as expressed by fewer Fluoro-Jade C-positive neurons) and local inflammation (lower expression of TNF-alpha) while improving the integrity of the blood-brain barrier (higher expression of claudin-1 and ZO-1). Given the nature of the study, we were not able to evaluate mobility, infarct size, or neurofunctional status in our animals. Instead, we analyzed neurodegeneration by immunofluoresence and expression of important biological markers within perilesional areas. Our data are in aggreement with previous experimental studies that demonstrated reduction in ischemic area and perilesional edema consequent to the administration of hyperoncotic albumin (7, 8).

The beneficial effects of albumin may be associated with different mechanisms, such as: increased antioxidant capacity, which reduces brain inflammation (7); the ability of albumin to transport endogenous and exogenous substances [fatty acids, glucose, electrolytes, among others (33)]; and its strong negative charge, which may stabilize membrane channels and restore the blood-brain barrier. A direct protective effect of albumin on endothelial cells, by prevention of apoptosis, may also contribute to the maintenance of membrane integrity (34, 35).

We found that hyperoncotic albumin increased tubular brush border damage, tubular vacuolization, desquamation of tubular cells, and cast formation in the kidney. This can be explained by the increase in intraglomerular oncotic pressure, with a reduction in the efficiency of ultrafiltration, as well as by podocyte lesion due to excess absorbed albumin, thus leading to increased apoptosis and local inflammation (36). Mitochondrial injury has also been reported as a result of excess protein reaching the tubular lumen (37), which may justify the more significant renal damage seen with higher concentrations of albumin in both models.

Clinical trials that evaluated the effects of albumin in acute ischemic stroke (9, 38) focused on perfusion (39), neurological score (38), infarct size (38, 40), and blood-brain barrier integrity (11). A multicenter randomized controlled trial has shown that treatment with intravenous albumin 25% at 2 g/kg within 5 h of acute ischemic stroke is not associated with improved outcome at 90 days (9). However, there are several differences between our experimental model compared to the aforementioned clinical study: (1) we started albumin infusion earlier (3 *vs*. 5 h after ischemic stroke); (2) the total amount of albumin was lower (0.42 g/kg for 5% ALB and 1.42 g/kg for 20% ALB *vs*. 2 g/kg); and (3) the total volume of albumin administered was lower (6.2 mL/kg for 5% ALB and 5.9 mL/kg for 20% ALB *vs*. 10 mL/kg). A single-center observational study in acute ischemic patients found that high-dose albumin (25%) was associated with better outcome compared to crystalloids (OR 1.81, 95% CI 1.11–2.94) (41), whereas a sub-analysis of the SAFE study (42) found higher mortality with low-dose albumin (4%, osmolality 260 mOsm/L) compared to normal saline. A retrospective single-center study (43) and a multicenter propensity-score study (44) found that high-dose albumin (25%) was associated with better neurological outcome *vs*. crystalloids.

On the basis of this evidence, a recent ESICM consensus on fluid therapy in neurointensive care recommended the use of crystalloids as preferred maintenance fluids in brain-injured patients and avoidance of high-dose (20–25%) albumin during the acute phase of stroke (3).

Based on our findings in the animal model of acute ischemic stroke used herein, we can hypothesize that a lower threshold for the amount and volume of isotonic albumin infused and early timing of administration can minimize both brain and kidney damage. This suggests that new randomized controlled trials taking into account this information are warranted before ruling out the use of albumin in acute ischemic stroke.

In the presence of brain-blood barrier damage, a higher concentration of albumin and greater infused volume may lead to increased albumin content in the interstitium, thus increasing oncotic pressure. Then, a greater shift of water and other macromolecules might occur in the interstitium, leading to greater extracellular matrix fragmentation and contributing to increased inflammation and cell apoptosis (38). This may explain the results of clinical studies (9, 10) in which higher concentrations and volumes of albumin were found to lead to brain damage.

### Limitations

The present study has some limitations. First, fluids were administered in a model of focal ischemic stroke induced by vessel thermocoagulation over the primary motor cortex. Our data cannot be extrapolated to other models of ischemic stroke with extensive brain damage (45). Second, fluid therapy was started 3 h after the ischemic injury; thus, the role of albumin and saline administration at different time points (particularly later in the course of stroke) has yet to be clarified. Third, neither animal behavior nor infarct size was evaluated due to the nature of our experimental design and timeline. Fourth, only male animals were used, both because they grow faster than female rats and to avoid any influence of hormonal fluctuation as a confounder on inflammatory parameters. The ARRIVE guidelines were followed throughout this investigation (14). However, the absence of female rats represents a limitation, according to recent studies (46). Fifth, In the current study, control group was used only for molecular biology analysis since we have used an practial approach comparing two different fluids with the one suggested by current guidelines in stroke condiction (3). Sixth, even though Wistar rats have more collateral vessels than Sprague-Dawley rats (47), which may be chosen for global cerebral ischemia instead of focal ischemia, the model used herein induced focal brain damage (17–19). Seventh, protein levels of different biomarkers were not analyzed, as modification in these parameters takes longer and would thus require prolonged follow-up. On the other hand, we were able to detect gene activation induced by different albumin concentrations and saline infusion. Further studies are needed to better evaluate the mechanisms of impact of different albumin concentrations in the brain and kidney.

## Conclusion

In the experimental model of focal ischemic stroke used herein, both iso-oncotic and hyperoncotic albumin solutions were associated with reduced expression of genes associated with neurodegeneration, brain inflammation, and damage to the blood–brain barrier compared to saline solution. However, hyperoncotic albumin resulted in greater kidney damage than iso-oncotic albumin. Only iso-oncotic albumin had a protective effect against brain and kidney damage in experimental focal ischemic stroke. Therefore, fluid therapy requires careful analysis of its impact not only on the brain, but also on the kidneys.

## Data Availability Statement

The datasets presented in this study can be found in online repositories. The names of the repository/repositories and accession number(s) can be found in the article/Supplementary Material.

## Ethics Statement

The animal study was reviewed and approved by Ethics Committee of the Carlos Chagas Filho Institute of Biophysics (CEUA 100/18), Federal University of Rio de Janeiro, Rio de Janeiro.

## Author Contributions

RM participated in the design of the study, carried out the experiments, performed data analyses, and drafted the manuscript. RM, GM, MO, and NR contributed to the study design and carried out the experiments. RM, NR, FC, MA, SA, and AS carried out the experiments and performed data analyses. MA and FC carried out the molecular biology analyses and contributed to the manuscript. RM, CT, PP-C, and RM-O performed the histological analyses and helped draft the manuscript. NR contributed to echocardiography analyses. RM, GM, MO, NR, FC, MA, SA, AS, CT, PP-C, RM-O, CR, PP, PR, and PS contributed to the study design, supervised the project, and helped write the manuscript. RM, PP, PR, and PS contributed to the study design, supervised the experimental work and statistical analysis, wrote the manuscript, and supervised the entire project. All authors read and approved the final manuscript.

## Conflict of Interest

The authors declare that the research was conducted in the absence of any commercial or financial relationships that could be construed as a potential conflict of interest.
