# Supplementary material for: Iso-Oncotic Albumin Mitigates Brain and Kidney Injury in Experimental Focal Ischemic Stroke
Source: Front Neurol. 2020 Sep 3;11:1001. doi: 10.3389/fneur.2020.01001 (PMC7494813; doi:10.3389/fneur.2020.01001)
Supplement: Supplementary file 1 [file Data_Sheet_1.docx]

**Additional File 1**

**Table S1: Fluid composition**

| **Composition** | **0.9% NaCl** | **20%ALB**  (CSL Behring) | **5%ALB**  (Baxalta) |
| --- | --- | --- | --- |
| **Osmolality** (mOsm/kg) | 308 | 263 | 250 |
| **Sodium caprylate** (mmol/L) |  | 16 | 4 |
| **Sodium *N*-acetyltryptophanate** (mmol/L) |  | 16 | 4 |
| **Na^+^** (mEq/L) | 154 | 140 | 140 |

| **Cl**^−^ (mEq/L) | 154 | — | — |
| --- | --- | --- | --- |

| **K^+^** (mEq/L) | — | — | — |
| --- | --- | --- | --- |
| **Ca^2+^** (mEq/L) | — | — | — |
| **Human albumin** (g/L) | — | 200 | 50 |

Na^+^, sodium; Cl^−^, chloride; Ca^2+^, calcium; ALB, albumin.

**Additional File 2**

**Table S2**: Forward and reverse oligonucleotide sequences of target gene primers

| **Gene** | **Primer** | **Primer sequences (5′-3′)** |
| --- | --- | --- |
| ***Brain*** | | |
| ZO-1 | Forward | caccacagacatccaaccag |
|  | Reverse | caccaaccactctcccttgt |
| Claudin-1 | Forward | ctgccaagaacctccaggag |
|  | Reverse | acttcgcaggtgagtgactg |
| TNF-α | Forward | TTGCTTCTTCCCTGTTCC |
|  | Reverse | CTGGGCAGCGTTTATTCT |
| BDNF | Forward | CTTTTGTCTATGCCCCTGCAGCCTT |
|  | Reverse | AGCCTCCTCTGCTCTTTCTGCTGGA |
| ***Kidney*** | | |
| KIM-1 | Forward | GAA GAA AAC AAT GGA TCA AGG GAT |
|  | Reverse | GGA GTG GAA ATG GCT CTA ATG AAC |
| IL-6 | Forward | CTCCGCAAGAGACTTCCAG |
|  | Reverse | CTCCTCTCCGGACTTGTGA |
| *36B4* | Forward | GGA TCA CTC AGG AGC AGG AG |
|  | Reverse | CTT GGC ACT CAA GAG GAA GG |

ZO-1, zonula occludens; TNF, tumor necrosis factor-α; BDNF, brain-derived neurotrophic factor; KIM, kidney injury molecule-1; IL, interleukin-6; *36B4*, acidic ribosomal phosphoprotein P0.

**Additional File 3**

Table 3: Arterial blood-gas analysis

| **Parameter** | **Group** | **INITIAL** | **FINAL** | **Time Effect** | **Group Effect** | **Time *vs.* Group Effect** |
| --- | --- | --- | --- | --- | --- | --- |
| **Arterial pH** | Saline | 7.32 [7.31-7.40] | 7.32 [7.25-7.33] | p=0.40 | p=0.14 | p=0.14 |
|  | 20%ALB | 7.34 [7.32-7.38] | 7.35 [7.27-7.38] |  |  |  |
|  | 5%ALB | 7.24 [7.22-7.34] | 7.31 [7.27-7.38] |  |  |  |
| **PaCO_2_**  **(mmHg)** | Saline | 32±8 | 38±11 | p=0.02 | p=0.09 | p=0.98 |
|  | 20%ALB | 36±3 | 42±9 |  |  |  |
|  | 5%ALB | 42±11 | 47±16 |  |  |  |
| **PaO_2_/FiO_2_**  **(mmHg)** | Saline | 387±104 | 419±81 | p=0.63 | p=0.48 | p=0.57 |
|  | 20%ALB | 361±90 | 329±102 |  |  |  |
|  | 5%ALB | 382±111 | 420±71 |  |  |  |
| **Anion gap**  **(mEq/L)** | Saline | 28.1±3.2 | 29.8±4.2 | p=0.72 | p=0.75 | p=0.15 |
|  | 20%ALB | 28.6±1.8 | 27.8±1.7 |  |  |  |
|  | 5%ALB | 28.6±5.3 | 26.9±3.9 |  |  |  |
| **Bicarbonate**  **(mEq/L)** | Saline | 18.6±3.3 | 19.2±2.6 | p=0.02 | p=0.14 | p=0.26 |
|  | 20%ALB | 20.5±2.1 | 21.9±3.3 |  |  |  |
|  | 5%ALB | 18.8±1.4 | 22.6±3.8^‡^ |  |  |  |

Data are shown as mean ± SD or median [interquartile rage]; 20%ALB: 20% albumin; 5%ALB: 5% albumin. Comparisons among groups at each time point (INITIAL and FINAL) were done by two-way ANOVA followed by Holm–Šídák’s multiple comparisons. PaCO_2_: arterial partial pressure of carbon dioxide; PaO_2_: arterial partial pressure of oxygen; FiO_2_: fraction of inspired oxygen. ^‡^ *vs* Initial (*p*<0.05).

| **Parameters** | **Groups** | **INITIAL** | **FINAL** | **Time Effect** | **Group Effect** | **Time *vs.* Group Effect** |
| --- | --- | --- | --- | --- | --- | --- |
| **V_T_ (mL/kg)** | Saline | 5.5±0.9 | 5.5±1.0 | p=0.25 | p=0.11 | p=0.23 |
|  | 20%ALB | 4.5±0.5 | 5.0±0.7 |  |  |  |
|  | 5%ALB | 4.9±0.5 | 5.3±0.9 |  |  |  |
| **RR (bpm)** | Saline | 67±7 | 67±7 | p=0.54 | p=0.52 | p=0.45 |
|  | 20%ALB | 70±6 | 69±6 |  |  |  |
|  | 5%ALB | 63±14 | 64±12 |  |  |  |
| **PPlat_RS_ (cmH_2_O)** | Saline | 10.1±2.5 | 10.9±2.6 | p=0.07 | p=0.11 | p=0.92 |
|  | 20%ALB | 13.7±4.0 | 15.0±5.6 |  |  |  |
|  | 5%ALB | 11.3±1.6 | 12.1±0.7 |  |  |  |
| **ΔP_RS_ (cmH_2_O)** | Saline | 7.1±2.5 | 7.9±2.6 | p=0.07 | p=0.11 | p=0.92 |
|  | 20%ALB | 10.7±4.0 | 12.0±5.6 |  |  |  |
|  | 5%ALB | 8.2±1.6 | 9.0±0.6 |  |  |  |
| **Energy_RS_ (mJ)** | Saline | 0.7±0.3 | 0.8±0.3 | p=0.05 | p=0.6 | p=0.58 |
|  | 20%ALB | 0.4±0.4 | 0.9±0.4 |  |  |  |
|  | 5%ALB | 0.7±0.2 | 0.9±0.2 |  |  |  |
| **Power_RS_ (mJ/min)** | Saline | 45.5±16.9 | 52.2±17.9 | p=0.09 | p=0.46 | p=0.66 |
|  | 20%ALB | 63.7±32.6 | 65.9±35.1 |  |  |  |
|  | 5%ALB | 46.8±23.2 | 57.6±12.9 |  |  |  |

**Additional File 4**

**Table S4. Ventilatory and Respiratory System Mechanics**

Data are shown as mean ± SD. Comparisons among groups at each time point (INITIAL and FINAL) were done by two-way ANOVA followed by Holm–Šídák’s multiple comparisons. Saline: 0.9% NaCl; ALB: albumin; V_T_: tidal volume; RR: respiratory rate; PPlat,_RS_: respiratory system plateau pressure; ΔP,_RS_: respiratory system driving pressure.
